# Effect of Extracellular Vesicles Derived From *Lactobacillus plantarum* Q7 on Gut Microbiota and Ulcerative Colitis in Mice

**DOI:** 10.3389/fimmu.2021.777147

**Published:** 2021-12-02

**Authors:** Haining Hao, Xinyi Zhang, Lingjun Tong, Qiqi Liu, Xi Liang, Yushan Bu, Pimin Gong, Tongjie Liu, Lanwei Zhang, Yongjun Xia, Lianzhong Ai, Huaxi Yi

**Affiliations:** ^1^ College of Food Science and Engineering, Ocean University of China, Qingdao, China; ^2^ Shanghai Engineering Research Center of Food Microbiology, School of Medical Instrument and Food Engineering, University of Shanghai for Science and Technology, Shanghai, China

**Keywords:** *Lactobacillus plantarum* Q7, extracellular vesicles, gut microbiota, inflammatory cytokines, ulcerative colitis

## Abstract

Probiotics plays an important role in regulating gut microbiota and maintaining intestinal homeostasis. Extracellular vesicles (EVs) derived from probiotics have emerged as potential mediators of host immune response and anti-inflammatory effect. However, the anti-inflammatory effect and mechanism of probiotics derived EVs on inflammatory bowel disease (IBD) remains unclear. In this study, the effect of *Lactobacillus plantarum* Q7-derived extracellular vesicles (Q7-EVs) on gut microbiota and intestinal inflammation was investigated in C57BL/6J mice. The results showed that Q7-EVs alleviated DSS-induced colitis symptoms, including colon shortening, bleeding, and body weight loss. Consumption of Q7-EVs reduced the degree of histological damage. DSS-upregulated proinflammatory cytokine levels including IL-6, IL-1β, IL-2 and TNF-α were reduced significantly by Q7-EVs (*p* < 0.05). 16S rRNA sequencing results showed that Q7-EVs improved the dysregulation of gut microbiota and promoted the diversity of gut microbiota. It was observed that the pro-inflammatory bacteria (Proteobacteria) were reduced and the anti-inflammatory bacteria (*Bifidobacteria* and *Muribaculaceae*) were increased. These findings indicated that Q7-EVs might alleviate DSS-induced ulcerative colitis by regulating the gut microbiota.

## Introduction

Inflammatory bowel disease (IBD), including ulcerative colitis (UC) and Crohn’s disease, are chronic inflammatory disorders of the gastrointestinal tract with a high incidence (1). Currently, the pathogenesis of IBD remains incompletely understood. The use of IBD drugs is severely limited by low efficacy, side effects and intolerance (2). It was reported that probiotics could alleviate IBD by maintaining normal gut microbiota, enhancing mucosal barrier function, and inhibiting exposure to inflammatory signals, which are expected to be a new treatment for IBD (3–5). The clinical effect and mechanism of probiotics on IBD are in progress (6).

Extracellular vesicles (EVs) are nano-scale membrane vesicles with phospholipid bilayer structures, which are secreted by almost all cells. At present, EVs have been isolated from animals (7, 8), plants (9) and microorganisms (10). Microorganism-derived EVs contain nucleic acids, proteins, and lipids, which play a crucial role in host metabolism and health (11). Microorganisms-host interactions mediated by EVs could result in various responses, which played potential roles as virulence factor delivery vehicles or inflammatory response modulators (12). For example, *Escherichia coli* Nissle 1917 derived EVs could mediate the anti-inflammatory and barrier protection effects in experimental colitis (13). EVs had been considered to be absent in Gram-positive bacteria due to their thick cell wall (11). Until 2009, Gram-positive bacteria-derived EVs was firstly confirmed (14). As Gram-positive bacteria, the functions of EVs derived from probiotics such as *Lactobacillus* and *Bifidobacteria* in immunity and intestinal diseases have attracted widespread attention. EVs derived from *Bifidobacterium longum* KACC 91563 alleviated food allergy through mast cell suppression (15). *Lactobacillus rhamnosus* GG derived EVs played a role in anti-proliferative on HepG2 cancer cells (16). *Lactobacillus reuteri* BBC3 derived EVs inhibited the inflammatory response mediated by activating macrophages, and played an important role in intestinal immune regulation (17). *Lactobacillus plantarum* derived EVs could be used as anti-inflammatory and immunomodulatory substances to improve inflammatory skin diseases and had the potential in treating antimicrobial-resistant pathogens (18, 19). *Lactobacillus* was considered to be the potential components involved with intestinal immune regulation (6). The effect of EVs derived from *Lactobacillus* on IBD has been rarely reported.

In our previous study, *Lactobacillus plantarum* Q7 (*L. plantarum* Q7) was isolated from the traditional Chinese fermented foods (20). It was observed that *L. plantarum* Q7 could exhibit activity against pathogenic bacteria such as *Escherichia coli, Listeria monocytogenes* and *Staphylococcus aureus*, which were associated with intestinal inflammation. The aim of this study was to explore the effect of *L. plantarum* Q7 derived EVs (Q7-EVs) on gut microbiota and ulcerative colitis in a DSS-induced colitis mouse model. It would provide a basis for probiotics to alleviate colitis from the perspective of EVs.

## Materials and Methods

### Isolation and Purification of Q7-EVs

*L. plantarum* Q7 (GenBank: CP019712-16) was isolated from traditional fermented yak yogurt made by herdsmen in Qinghai province, China (21). The strain was cultured in MRS medium at 37°C overnight. Q7-EVs were isolated from the culture supernatants using ultrafiltration combined with ultracentrifugation (22). In brief, the culture supernatants were centrifuged at 8000×*g* for 30 min to remove cell debris and impurities. After filtered by 0.22 μm filter, the supernatant was concentrated using centricon-plμs-70 100-kDa ultrafiltration tube (Millipore) and ultracentrifuged at 100000×*g* for 2 h (Hitachi, Ltd., Tokyo, Japan). The precipitate was aspirated gently by adding PBS and centrifuged at 100000×*g* for 1 h, then resuspended the pellet with PBS. Q7-EVs were collected and stored at -80°. The morphology and the size distribution of Q7-EVs were observed by Transmission electron microscope (H-7650, Hitachi, Ltd., Tokyo, Japan) and Dynamic light scattering (Nanotrac Wave II, Microtrac, Inc., Montgomeryville, USA).

### Animals Model

4-5 weeks old SPF male C57BL/6J mice were purchased from the Laboratory Animal Breeding Center of Pengyue (Jinan, China). Mice were housed in an animal care facility and provided with sufficient food (Beijing Keao Xieli Feed Co., Ltd., Beijing, China) and water. Colitis was induced by 3.5% dextran sulfate sodium (DSS, Yeasen Biotech, Shanghai, CN) administration in the drinking water (23) and the mice were treated Q7-EVs by gavage (23, 24). The mice were acclimated for a week and randomly divided into four groups: control group (PBS, daily), DSS+PBS group (PBS, daily), DSS+10 μg Q7-EVs group (0.5 mg/kg body weight Q7-EVs, daily), DSS+20 μg Q7-EVs group (1 mg/kg body weight Q7-EVs, daily). The mice in control group were administered untreated drinking water for 18 days. The DSS group and treatment group were pre-garaged with PBS or Q7-EVs for 10 day and received 3.5% DSS in drinking water from 11^th^ day to 18^th^ day. All experimental processes were approved by Animal Ethics Committee of Ocean University of China (permission number: spxy20200720215).

### Evaluation of Disease Activity Index (DAI) and Spleen Index

Body weight, stool characteristic and rectal bleeding were recorded every two days. DAI scores were calculated according to previously described methods (25–27). The details of each score were listed in **Supplementary Materials** (**Table S1**). The weight of spleen was determined, and the spleen index was defined as the ratio of spleen weight to body weight as previous report (28).

### Histological Analysis

Colon tissue was fixed in 4% paraformaldehyde, dehydrated with gradient alcohol solution and embedded in paraffin. Paraffin-embed samples were cut into continuous longitudinal sections, and then stained by H&E. The histological scoring was measured according to the criteria listed in **Table S2** (29).

### Enzyme-Linked Immunosorbent Assay

According to the manuscript’s protocol, the expression level of IL-6, IL-1β, and TNF-α in the serum was determined by ELISA kit (Nanjing Jiancheng Biotechnology, Nanjing, China). Multiskan FC (Thermo Scientific, Waltham, MA, USA) was used to detect the absorbance of each sample at 450 nm.

### RT-qPCR Analysis

The colon tissue was put into Trizol reagent and homogenized using a tissue grinder (Wuhan Sevelco Co., Ltd., Wuhan, China). 1 μg RNA was reverse transcribed *via* a high-capacity cDNA reverse transcription kit (Applied Biosystems, Foster City, USA). Genes were quantitated using the StepOnePlus real-time PCR instrument (Applied Biosystems, Foster City, USA). The specific primers used were shown in **Table S3**. The 2^-ΔΔCt^ method was utilized to normalize the expression results. The glyceraldehyde 3-phosphate dehydrogenase (GAPDH) was used as the housekeeper gene to normalize each mRNA.

### 16S rRNA Gene Sequencing

Fecal DNA extraction and high-throughput sequencing were performed by Personal Biotechnology Co., Ltd (Shanghai, China). Briefly, the Fast DNA SPIN extraction kit (MP Biomedicals, Santa Ana, CA, USA) was used to extract genomic DNA from mice feces. The highly variable V3-V4 region of the bacterial 16S rDNA gene was selected for amplification. The library was qualified based on the TruSeq Nano DNA Library Prep Kit from Illumina, and Illumina NovaSeq platform was used for paired-end sequencing. The filtered sequences were clustered as operational taxa (OTU) using the UCLUST algorithm with a similarity threshold of 97%. The OTU abundance and the six-level taxonomic classification from phylum to species were obtained for analysis.

### Statistical Analyses

The experimental data were analyzed by GraphPad Prism version 7.0 and SPSS 25.0 statistical software. Comparisons of various anatomical measurements by two-way analysis of variance (ANOVA), followed by Fisher’s LSD *post hoc* tests. PCA and PCoA analyzed were used R language pack. The linear discriminant analysis (LDA) was applied to identify the differences of microbiota compositions between different groups by using an LDA score threshold of >3.5.

## Results

### Characterization of Q7-EVs

**Figure 1:**
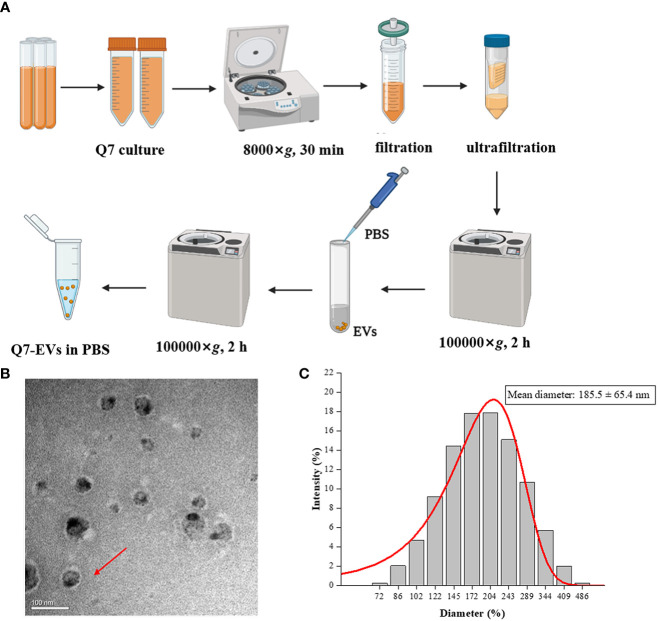
Characterization of Q7-EVs. **(A)** Isolation and purification procedures of Q7-EVs. **(B)** Transmission electron microscopy images of Q7-EVs. **(C)** Size distribution of Q7-EVs.

EVs were isolated from the supernatant of *L. plantarum* Q7 by ultrafiltration combined with ultracentrifugation **(** **Figure 1A** **)**. The Q7-EVs were characterized by Transmission electron microscope and Dynamic light scattering. The results showed that Q7-EVs were double-layer membrane-enclosed nanoparticles with spherical morphology (**Figure 1B**). It was observed that Q7-EVs ranged from 70 nm to 500 nm in size **(** **Figure 1C** **)**, the mean size was 185.5 ± 65.4 nm, which was consistent with the size of previous reports (30).

### Effect of Q7-EVs on DSS-Induced Colitis

**Figure 2:**
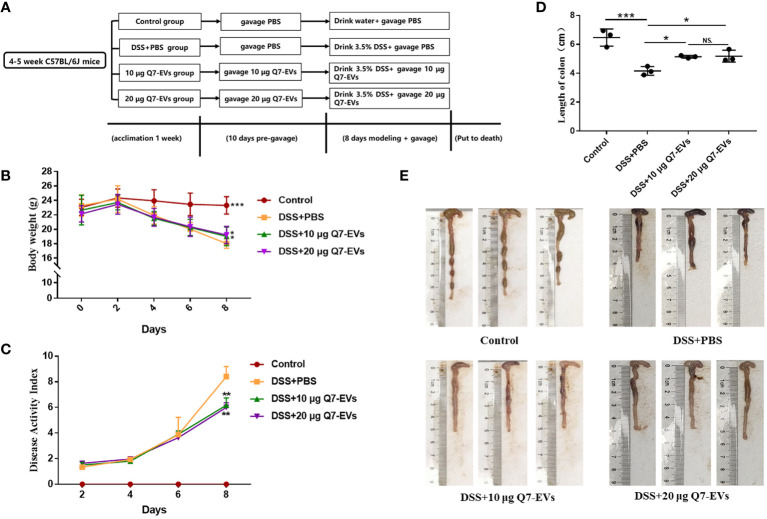
The effect of Q7-EVs on DSS-induced colitis in C57BL/6J mice. **(A)** Overall experimental design. **(B)** Body weight. **(C)** Disease activity index. **(D, E)** Representative photograph and statistical analysis of colon length. Values were means ± SD (*p < 0.05, **p < 0.01, ***p < 0.001, NS. represents no significant difference).

The effect of Q7-EVs on DSS-induced colitis was evaluated in C57BL/6J mice (**Figure 2A**) (31). The body weight, DAI, length of the colon, and colonic histomorphology were analyzed. Compared with the control group, the weight loss could be slowed by Q7-EVs intervention since the 6^th^ day. The weight loss of 22.6% was observed in the DSS+PBS group, while the weight loss of 12.8% and 12.6% were found in the 10 μg and 20 μg Q7-EVs group on the 8^th^ day, respectively. The weight loss could be slowed by Q7-EVs intervention with no dose correlation (**Figure 2B**). DAI scores of Q7-EVs (10 μg and 20 μg) treated mice were less than that of DSS+PBS group mice on the 8^th^ day (*p* < 0.05) (**Figure 2C**). Colon length was regarded as a key indicator to assess the severity of acute colitis induced by DSS (31, 32). It was confirmed that Q7-EVs treatment improved the shortening of the colon significantly (*p* < 0.05) (**Figures 2D, E** **)**. The colon length in the DSS+PBS group was 4.16 ± 0.24 cm. After treatment with Q7-EVs, the average length of the colon was observed to be 5.14 ± 0.07 and 5.19 ± 0.34 cm, respectively. The spleen is one of important immune organ of the body, which exerted a regulatory effect on the immunity. It was found that the Q7-EVs reduced the spleen index of DSS-induced colitis mouse model (**Figure S1**).

**Figure 3:**
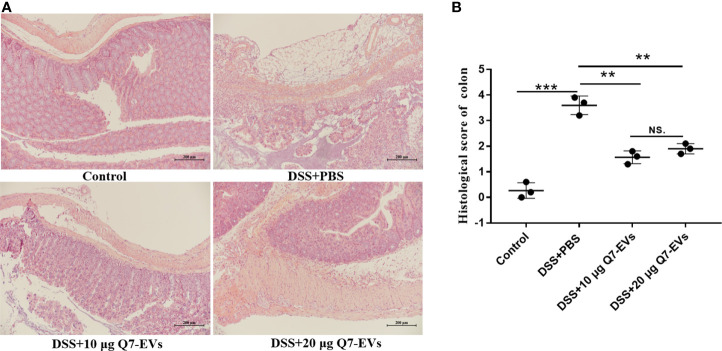
Pathophysiological analysis of colon sections. **(A)** Representative microscopy images of H&E-stained colonic sections. **(B)** Histological scoring of the colon section. Data were expressed as means ± S.D (**p < 0.01, ***p < 0.001, NS. represents no significant difference).

H&E staining showed that DSS treatment caused damage to the colon structure, while the pathological features were improved by oral Q7-EVs (**Figure 3A**). It suggested that Q7-EVs could reduce the colonic pathology score of mice and alleviate colitis in mice significantly (**Figure 3B**).

### Effect of Q7-EVs on the Expression of Inflammatory Cytokines

**Figure 4:**
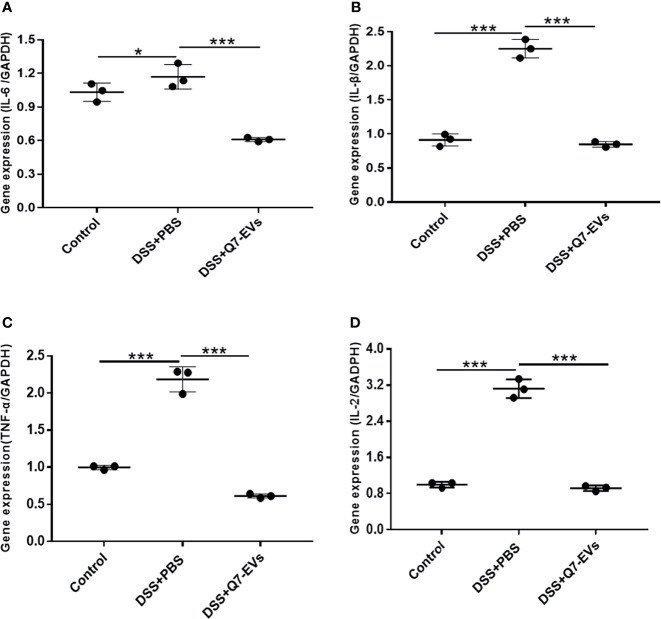
The effect of Q7-EVs on the mRNA Expression level in colon tissue. **(A)** IL-6, **(B)** TNE-α, **(C)** IL-β, **(D)** IL-2. Data were expressed as means ± S.D (*p < 0.05, ***p < 0.001).

It was observed that there was no significant difference in the effects of two doses of Q7-EVs (10 μg and 20 μg) on the body weight, colon length and colonic pathology score. Therefore, 10 μg Q7-EVs was selected in the following experiments. It was reported that the increase of pro-inflammatory cytokines was a hallmark of DSS-induced colitis (33). The level of inflammatory cytokines in colon tissues was detected (34). It was found that the expression levels of IL-6, IL-1β, TNF-α, and IL-2 were increased in the colon tissue of the DSS+PBS group mice. In contrast, these cytokines were significantly reduced in the colon under the intervention of Q7-EVs (**Figure 4**). A similar trend was found for the determination of IL-6, IL-1β, and TNF-α in serum (**Figure S2**).

### Effect of Q7-EVs on Intestinal Microbiota Composition in Colitis Mice

**Figure 5:**
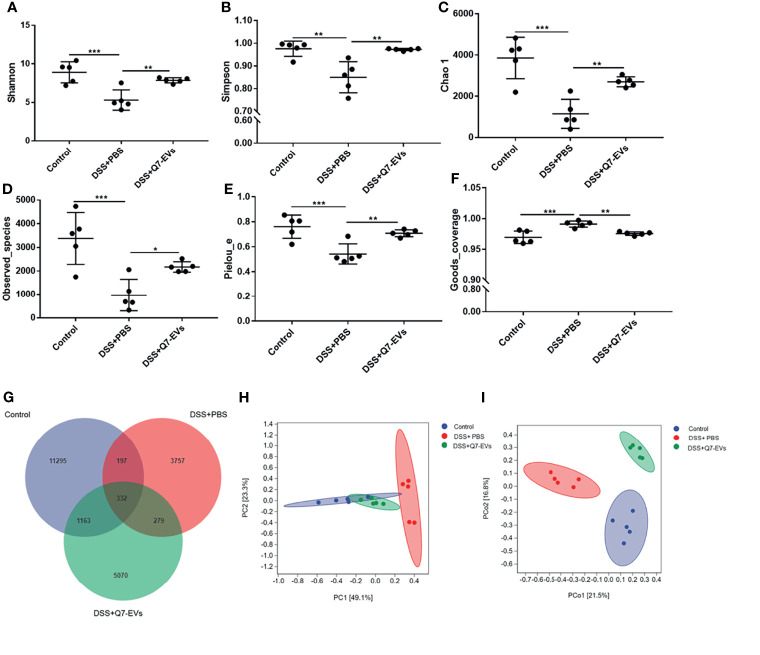
The effect of Q7-EVs on gut microbiota in colitis mice. Alpha diversity analysis included **(A)** Shannon index, **(B)** Simpson index, **(C)** Chao 1 index, **(D)** Observed_species, **(E)** Pielou_e, and **(F)** Goods_coverage. Comparative analysis of microbiota composition included **(G)** Venn diagram of OTU, **(H)** PCA analysis, and **(I)** PCoA analysis. Data were expressed as means ± S.D (*p < 0.05, **p < 0.01, ***p < 0.001).

It was found that microbial richness, evenness and diversity were significantly decreased in the DSS+PBS group in comparison with that of the control group, whereas Q7-EVs could restore the species diversity, richness and community evenness (**Figures 5A–F**).

**Figure 6:**
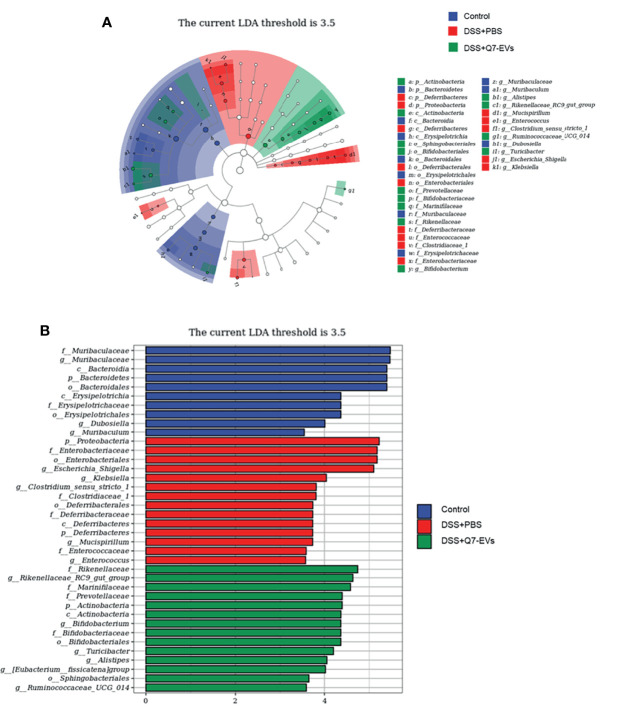
The effect of Q7-EVs on gut dominant microorganisms. **(A)** Cladogram displayed the taxonomic tree of differentially abundant taxa by LEfSe analysis on genus. **(B)** Distribution histogram of gut dominant microorganisms on genus.

Venn diagram was used to display the composition information of species in each group. It was observed that 332 OTUs overlapped in all three groups, 197 OTUs were present in both the control and DSS+PBS groups, 279 OTUs in the DSS+PBS and Q7-EVs groups, and 1163 OTUs in the control and DSS+Q7-EVs groups (**Figure 5G**). Compared with the DSS+PBS group, intestinal microbial composition in the DSS+Q7-EVs group was more similar to the control group. The results showed that the gut microbiota of the DSS+PBS group was significantly different from that of the control group, and Q7-EVs alleviated the shift of gut microbiota induced by DSS (**Figures 5H, I** **)**. LEfSe analysis was demonstrated that 37 key phylotypes were determined as distinguished biomarkers on the genus (**Figure 6A**). The control group had 10 dominant microorganisms, the DSS+PBS group and the Q7-EVs group had 14 dominant microorganisms, respectively (**Figure 6B**).

**Figure 7:**
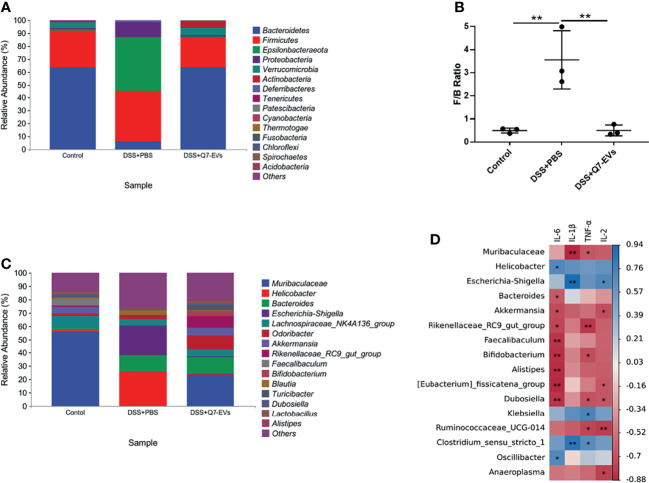
The effect of Q7-EVs on the community structures of gut microbiota and its relationship with inflammatory cytokines. **(A)** Microbial community bar plot by phylum. **(B)** The ratio of Firmicutes/Bacteroidetes. **(C)** Microbial community bar plot by genus. **(D)** Correlation matrix between the microbiota and inflammatory cytokines. Data were expressed as means ± S.D **p < 0.01.

The composition of the gut microbiota at the phylum level was shown in **Figure 7A**. Compared with the control group, Bacteroidetes, and Verrucomicrobia in the DSS+PBS group were significantly decreased, while Proteobacteria, Epsilonbacteraeota were increased significantly. These changes of gut microbiota were recovered in the Q7-EVs group. The abundance of Proteobacteria was decreased in the DSS+Q7-EVs group. The Firmicutes/Bacteroidetes (F/B) ratio in the DSS+PBS group was higher than that of the control group, and was restored after the intervention of Q7-EVs (**Figure 7B**), which could be used as a biomarker for intestinal inflammation (31). The difference of gut microbiota in each group at the genus level was shown in **Figure 7C**. Compared with the DSS+PBS group, *Bifidobacterium*, *Rikenellaceae_RC9_gut_group*, *Akkermansia*, *Muribaculaceae*, *Lactobacillus* and *Alitipes* in the DSS+Q7-EVs group were recovered to the level of the control group **(** **Figure S3** **)**.

Correlation analysis was conducted to determine the relationship between the gut microbiota and inflammatory cytokines. Proteobacteria (*Helicobacter*, *Escherichia-shigella*, *Klebsiella*, *Oscillibacter*) and Firmicutes *(Clostridium_sensu_stricto_*1) were positively correlated with inflammatory cytokines. *Muribaculaceae*, *Bacteroides*, *Akkermansia*, *Rikenellaceae_*RC9*_gut_group*, *Faecalibaculum*, *Bifidobacterium*, *Alistipes*, *Eubacteriu_fissicatena_ group*, *Dubosiella*, *Ruminococcaceae_*UCG-014 and *Anaeroplasma* were negatively correlated with inflammatory cytokines (**Figure 7D**). Combined with the LEfSe analysis, the dominant microorganisms in the DSS+Q7-EVs group contained more anti-inflammatory bacteria.

## Discussion

UC is a common type of IBD with an increasing incidence in recent years (28). Probiotics can protect the intestinal mucosal barrier, inhibit intestinal inflammation, and maintain the balance of the gut microbiota structure (31), which may offer an alternative or adjuvant strategy to UC therapy (35). Probiotics-derived EVs has attracted increasing attention due to the potential for the treatment of multiple diseases, such as inflammation (17, 18, 22), bacterial infection (19) and obesity (36). *L. plantarum* Q7 was isolated from Chinese traditional food in our previous study (21). The potential probiotic character of *L. plantarum* Q7 had been investigated in the view of bacteriocin. In this study, the effect of *L. plantarum* Q7 on DSS-induced colitis was explored from the perspective of EVs.

It was found in this study that Q7-EVs could ameliorate DSS-induced colitis *via* regulating gut microbiota in C57BL/6J mice. The changes of inflammatory cytokine in mice serum and colon tissue were explored. Cytokines such as IL-6, IL-1β, IL-2 and TNF-α were involved in the inflammatory response (37–40). As expected, the Q7-EVs ameliorated the immune response and restored the expression of the cytokines. NF-κB was the main inflammatory pathway in the transcription of pro-inflammatory cytokines (such as IL-6, IL-1β, and TNF-α) (28). TLR4 and MyD88 played a critical role in the development of DSS-induced colitis (41). In our study, the gene expression of TLR4 and MyD88 in the colon was determined. The results showed that oral Q7-EVs down-regulated TLR4 and MyD88 gene expression compared with DSS-induced colitis mice **(** **Figure S4** **)**. After binding to the ligand, MyD88-dependent signal transduction could lead to phosphorylation of NF-κB, thereby regulating the levels of transcription factors of IL-1β, IL-6, and TNF-α (24). It was speculated that Q7-EVs improved colitis by regulating the TLR4-MyD88-NF-κB pathway.

Gut microbiota played an important role in intestinal inflammation, which was related to the etiopathogenesis of UC. Different microbes participated in either pro-inflammatory or anti-inflammatory processes (42). It was reported that EVs could play a role in host-microbe responses and regulate the gut microflora (43). Proteobacteria was reported to be the main phylum of pathogenic bacteria, which promoted the production of excessive pro-inflammatory cytokines and related to IBD pathogenesis (24, 42). *Akkermansia* was a confirmed probiotic with anti-inflammatory effect by improving neutrophil infiltration and alleviating intestinal inflammation (44–46). *Bifidobacteria* could exert an anti-inflammatory effect through induction of intestinal IL-10 and protect from Th1-driven inflammation (42). *Muribaculaceae* could inhibit the CD8^+^ T cell activation to tolerate the immunity stimulation and present a negative correlation with inflammation status (47). In our study, the correlation between gut microbiota and inflammatory cytokines (IL-6, IL-1β, TNF-α and IL-2) was analyzed at the genus level. It was found that Q7-EVs increased the number of *Akkermansia*, *Bifidobacteria*, *Muribaculaceae* and *Lactobacillus*. On the other hand, Q7-EVs reduced the number of Proteobacteria, Deferribacteres and Epsilonbacteraeota. These results suggested that Q7-EVs might improve inflammation by regulating the gut microbiota in DSS-induced colitis mouse model. *Lactobacillus and Bifidobacteria* are common probiotics in the intestine, which help to inhibit harmful bacteria and improve gastrointestinal barrier function (48). Proteobacteria were considered as harmful bacteria in UC (49, 50). These findings indicated that Q7-EVs relieved DSS-induced colitis by increasing the beneficial bacteria and reducing the harmful bacteria in the intestine. Though it was confirmed that Q7-EVs could regulate the gut microbiota and alleviate DSS-induced colitis, the specific functional components and the mechanism of probiotic-derived EVs alleviating UC remained to be investigated in the future.

In conclusion, EVs derived from *L. plantarum* Q7 could regulate the intestinal microbiota and ameliorate DSS-induced colitis in C57BL/6J mice. *L. plantarum* Q7 might exert the probiotic function mediated by its extracellular vesicles. These findings provided a novel perspective to explore the function mechanism of probiotic and develop a potential therapeutic treatment of IBD.

## Data Availability Statement

The original contributions presented in the study are publicly available. This data can be found here: https://www.ncbi.nlm.nih.gov/bioproject/, PRJNA778054.

## Ethics Statement

The animal study was reviewed and approved by Animal Ethics Committee of Ocean University of China (permission number: spxy20200720215).

## Author Contributions

Conceptualization, HY and LA. Methodology, HH, XZ, and TL. Formal analysis, HH, LT, and XL. Software, HH and QL Supervision, HY and LZ. Data curation, HH and YX. Writing-original draft preparation, HH, XZ, and LT. Writing-review and editing, HY, TL, and PG. Visualization, YB and YX. Project administration, HY. Funding acquisition, HY. All authors contributed to the article and approved the submitted version.

## Funding

This work was supported by National Natural Science Foundation of China (No. 32172180, 31771988) and Key Program of Natural Science Foundation of Shandong Province in China (No. ZR2020KC009).

## Conflict of Interest

The authors declare that the research was conducted in the absence of any commercial or financial relationships that could be construed as a potential conflict of interest.

## Publisher’s Note

All claims expressed in this article are solely those of the authors and do not necessarily represent those of their affiliated organizations, or those of the publisher, the editors and the reviewers. Any product that may be evaluated in this article, or claim that may be made by its manufacturer, is not guaranteed or endorsed by the publisher.
