# Supplementary material for: Effect of Extracellular Vesicles Derived From Lactobacillus plantarum Q7 on Gut Microbiota and Ulcerative Colitis in Mice
Source: Front Immunol. 2021 Dec 2;12:777147. doi: 10.3389/fimmu.2021.777147 (PMC8674835; doi:10.3389/fimmu.2021.777147)
Supplement: Supplementary file 1 [file DataSheet_1.docx]

**SUPPLEMENTARY MATERIALS**

| **Score** | **Body Weight** | **Stool characteristic** | **Rectal bleeding** |
| --- | --- | --- | --- |
| 0 | No Body weight loss or body weight loss less than 1% | Normal stool | Negative hemoccult |
| 1 | Body weight loss between 1% and 5% | Soft stools | Negative hemoccult |
| 2 | Body weight loss between 5% and 10% | Very soft stools | Small amounts of blood-streaked feces |
| 3 | Body weight loss between 11% and 20% | Loose stools | Conspicuous blood-wrapped feces |
| 4 | Body weight loss more than 20% | Watery stools | Visible rectal bleeding |

**Table S1** Scoring standard of disease activity index

The DAI score equals the sum of body weight, stool characteristic and rectal bleeding.

**Table S2** Scoring standard of histological injury in colon

| Score | [Evaluation](javascript:;) i[tem](javascript:;) | | | | | |
| --- | --- | --- | --- | --- | --- | --- |
|  | Degree of inflammatory cell infiltration | The muscularis and serosal layers | | Destruction of intestinal crypts | | [Extent](javascript:;) [of](javascript:;) [disease](javascript:;) (%) |
| 0 | NO | | NO | NO | NO | |
| 1 | light | | mucous layer | 1/3 of base crypts destroyed | 1-25 | |
| 2 | medium | | [submucosa](javascript:;) | 2/3 of base crypts destroyed | 26-50 | |
| 3 | [serious](javascript:;) | | The muscularis and serosal layers | Only intact surface epithelium | 51-75 | |
| 4 | - | | - | All crypts and epithelium were destroyed | 76-100 | |

**Table S3** Primers used for real-time PCR

| Gene | Primer |
| --- | --- |
| IL-6-F | ACTTCCATCCAGTTGCCTTCTTGG |
| IL-6-R | TTAAGCCTCCGACTTGTGAAGTGG |
| TNF-α-F | GCGACGTGGAACTGGCAGAAG |
| TNF-α-R | GCCACAAGCAGGAATGAGAAGAGG |
| IL-1β-F | TCGCAGCAGCACATCAACAAGAG |
| IL-1β-R | TGCTCATGTCCTCATCCTGGAAGG |
| IL-2-F | GCAGCTCGCATCCTGTGTCAC |
| IL-2-R | CTGCTGTGCTTCCGCTGTAGAG |
| MyD88-F | GCTAGAGCTGCTGGCCTTGTTAG |
| MyD88-R | TCTCGGACTCCTGGTTCTGCTG |
| TLR-4-F | ACAAGGCA TGGCA TGGCTTACAC |
| TLR-4-R | TGTCTCCACAGCCACCAGA TTCTC |
| GAPDH-F | GGTTGTCTCCTGCGACTTCA |
| GAPDH-R | TGGTCCAGGGTTTCTTACTCC |


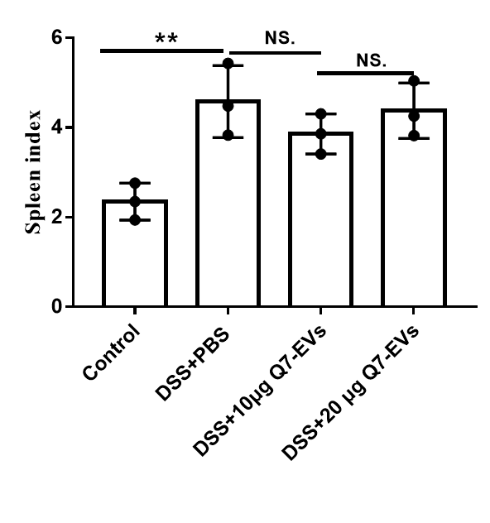


**Figure S1** Mouse spleen index with DSS-induced colitis treated with Q7-EVs. Data were expressed as means ± S.D. (**p* < 0.05, ** *p* < 0.01, *** *p* < 0.001)


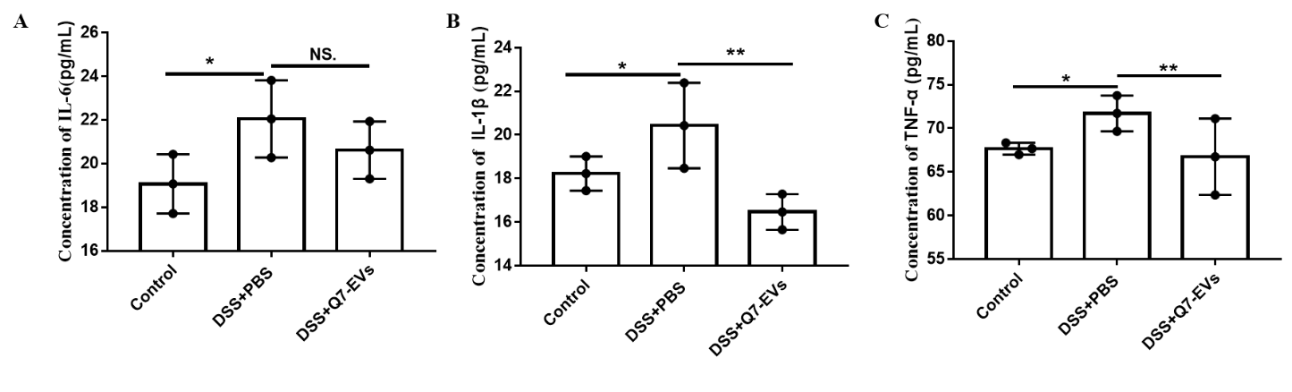


**Figure S2** The effect of Q7-EVs on the inflammatory cytokines levels in serum. (A) IL-6 (B) TNE-α (C) IL-β. Data were expressed as means ± S.D (**p* < 0.05, ** *p* < 0.01, *** *p* < 0.001).


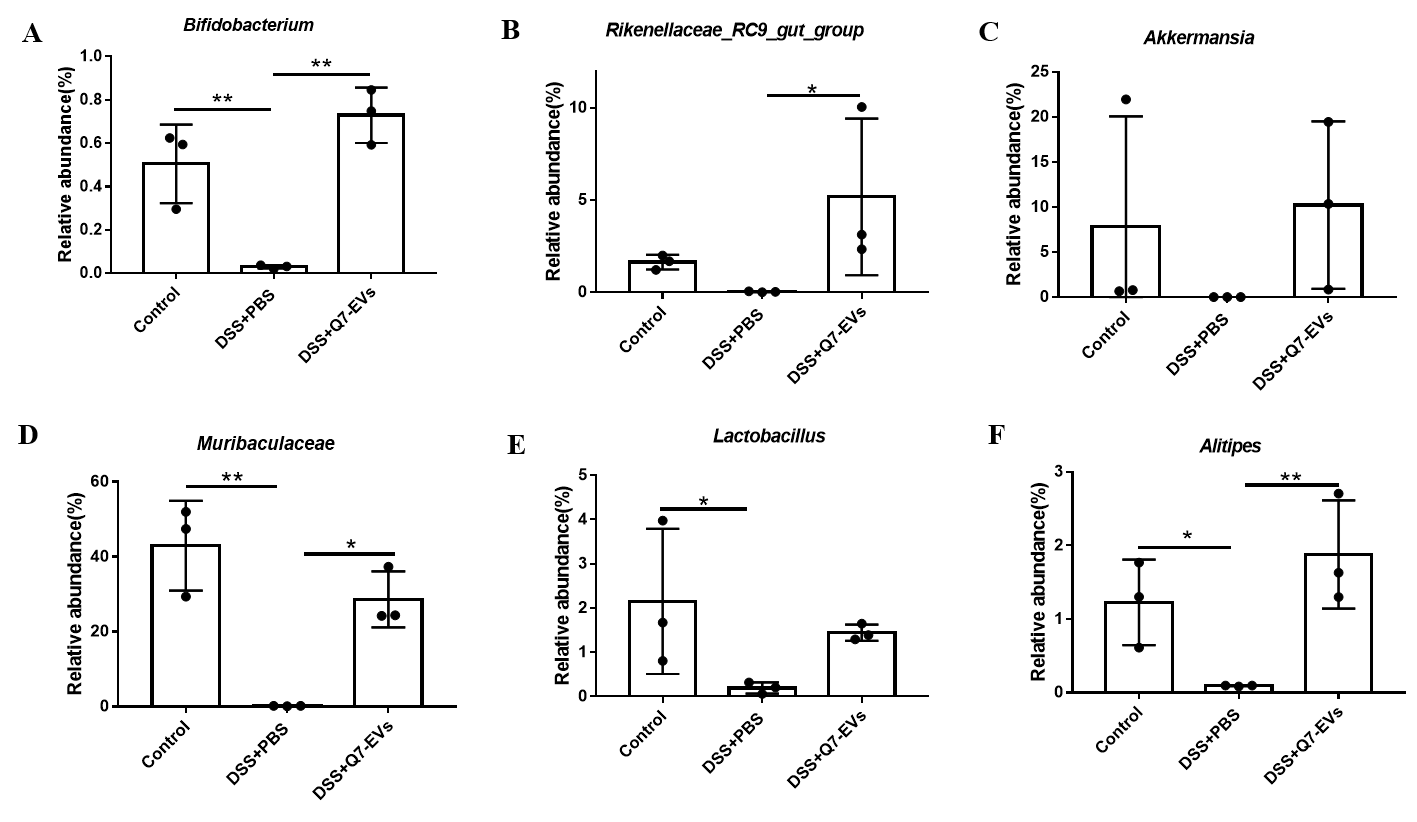


**Figure S3** The difference of gut microbiota at the genus level. (A) *Akkermansia* (B) *Rikenellaceae_RC9_gut_group* (C) *Bifidobacterium* (D) *Muribaculaceae* (E) *Lactobacillus* (F) *Alitipes.*


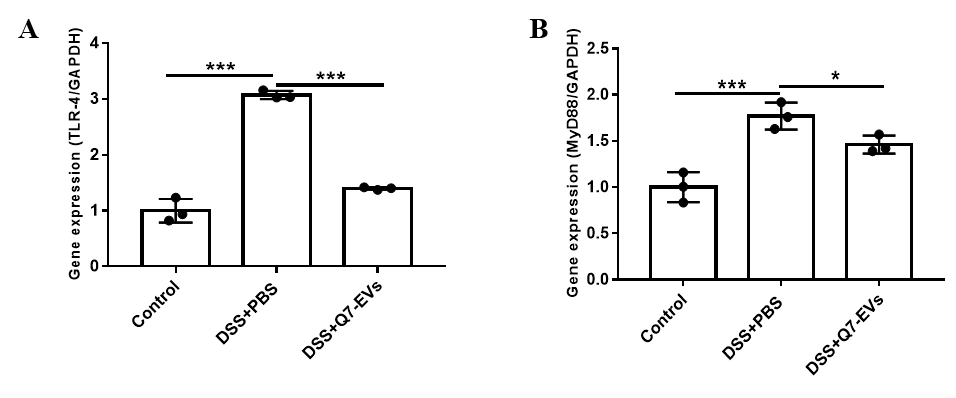


**Figure S4** mRNA Expression level in colon tissue. (A) TLR-4; (B)MyD88.
